# Feasibility, acceptability and preliminary evaluation of a user co-facilitated psychoeducational programme: a feasibility proof-of-concept randomised control trial

**DOI:** 10.1186/s12888-024-06015-4

**Published:** 2024-09-16

**Authors:** Tatiana Skliarova, Mariela L. Lara-Cabrera, Hege Hafstad, Audun Havnen, Sverre Georg Saether, Øyvind Salvesen, Jonas Vaag, Terje Torgersen

**Affiliations:** 1 Department of Mental Health, Faculty of Medicine and Health Sciences, Norwegian University of Science and Technology (NTNU), Trondheim, Norway; 2 Nidelv Community Mental Health Centre, Department of Mental Healthcare, St. Olavs Hospital, Trondheim University Hospital, Trondheim, Norway; 3 Vårres Regional User-Led Center Mid-Norway, Trondheim, Norway; 4 Nidaros Community Mental Health Center, Division of Psychiatry, St. Olavs University Hospital, Trondheim, Norway; 5 Department of Psychology, Norwegian University of Science and Technology (NTNU), Trondheim, Norway; 6 Blue Cross Lade Addiction Treatment Centre, Trondheim, Norway; 7 Department of Public Health and Nursing, Norwegian University of Science and Technology, Trondheim, Norway; 8 Department of Psychology, Inland University of Applied Sciences, Lillehammer, Norway

**Keywords:** Adult ADHD, Co-creation, Feasibility, Group treatment, Patient education, Patient satisfaction, Peer co-led, Psychoeducation, Randomised controlled trial, Self-efficacy, Self-management, User-centred design

## Abstract

**Background:**

Mental health settings are increasingly using co-facilitation of educational group interventions in collaboration with patient partners and service users. However, despite promising results, limited information is available regarding the feasibility and satisfaction levels of these programmes among adults newly diagnosed with attention-deficit hyperactivity/impulsivity disorder (ADHD). Hence, this study aimed to determine the feasibility, acceptability, and preliminary effects of a user co-facilitated psychoeducational group programme for adults diagnosed with ADHD.

**Methods:**

This feasibility proof-of-concept randomised controlled trial recruited outpatients from a Norwegian community mental health centre. Outpatients randomised to the intervention group (IG) received a psychoeducational programme supplementing Treatment As Usual (TAU), while the control group received TAU. Feasibility was determined by the acceptance rate, adherence rate, and dropout rate. Acceptability was measured with the Client Satisfaction Questionnaire and a 3-item scale measuring satisfaction with the received information. To test the preliminary effects, self-efficacy, symptom severity, and quality of life were measured at baseline and pre- and post-intervention.

**Results:**

Feasibility was demonstrated; most of the patients were willing to enrol, participants attended 82% of the psychoeducational programme, and only 13% dropped out of the study. The between-group analyses revealed that the IG reported significantly greater mean satisfaction than the CG. Moreover, the intervention group was more satisfied with the information they received during the psychoeducational programme. Concerning the preliminary effects, the linear mixed model showed improvement in quality of life (the subscale relationship); however, other patient-reported outcomes did not show improvements.

**Conclusions:**

This proof-of-concept randomised controlled trial supports the feasibility and acceptability of the user co-facilitated psychoeducational programme for patients newly diagnosed with ADHD in an outpatient setting. While preliminary findings indicate promise in enhancing patient-reported outcomes, a larger study is warranted to assess the intervention’s effectiveness rigorously.

**Trial registration:**

NCT03425, 09/11/2017.

## Introduction

Attention-deficit hyperactivity/impulsivity disorder (ADHD) is a long-term neurodevelopmental disorder [1] that can have a negative impact on adults and children [2, 3]. Among adults, the average prevalence rate has been estimated at 2.8% [4]. The main symptoms of the disorder include inattentiveness, hyperactivity, and impulsivity. In addition, individuals with ADHD often experience the burden of psychiatric comorbidity [5–9], and research findings indicate that comorbidity rates with disorders including anxiety and depression are high among adult patients with ADHD [10, 11]. ADHD is also associated with functional impairments that affect work performance, psychosocial functioning [12, 13], educational functioning [1, 14] and quality of life [15]. Moreover, adults with ADHD have fewer psychological protective factors, such as self-management skills [16, 17].

Self-efficacy is defined as a person’s belief in their ability to control the complex demands of the environment through adaptive actions [18]. It plays an essential protective role in managing stressful situations [19] and may be important for improving patient participation in treatment. In addition, among patients with various chronic diseases, self-efficacy is positively associated with quality of life [20]. An increasing number of non-pharmacological interventions aim to improve the level of self-efficacy in adults with mental disorders [21–24]. Furthermore, peer-based and peer co-facilitated interventions can improve self-efficacy via vicarious experience and activating coping skills [25].

While different non-pharmacological treatments are well-documented for adult ADHD, there is potential for further improvement by incorporating psychoeducational programmes into ADHD treatment pathways. From a mental healthcare perspective, educational interventions represent one way to help newly diagnosed patients acquire the skills needed to independently self-manage their condition. These interventions can effectively support them in accepting, adjusting to and living with their ADHD symptoms [26]. Recent studies have demonstrated that outpatients diagnosed with ADHD require and are interested in learning about different aspects of their diagnosis [27–29]. For patients with ADHD, psychoeducational programmes help them acquire knowledge and understand their disorder and the impairments it entails [30]. By providing appropriate information, an educational programme may facilitate patients’ acceptance of the disorder and ameliorate negative emotions, such as guilt [30].

Psychoeducational interventions are treatment approaches that can be delivered during the treatment waiting time [31, 32], and in conjunction with standard clinical care [33], such as pharmacological treatment and psychotherapeutic sessions with psychologists. The interventions can be delivered individually or as a group intervention [30, 34–37] and may involve collaboration with peers and former patients [31, 33, 38–42]. According to the review conducted in 2024 [43], psychoeducational interventions playing a valuable role in supporting adults with ADHD by providing information, coping skills, and social support. Emerging evidence suggests that psychoeducation reduces the levels of core symptoms [34]; improves quality of life and self-efficacy [16, 35], enhances peer support [44]; and improves self-management skills, psychosocial functioning and health outcomes [32].

Professionals carry out and facilitate most educational group programmes [45–49], but emerging evidence suggests that educational groups co-facilitated in collaboration with service users improve self-management skills [32] and the usage of health services [25]. Co-facilitated educational group interventions within a community mental health centre resulted in improvements in knowledge about treatment options [31], increased patient activation, improved attendance [32, 50] and reduced dropout rates [39]. Findings also indicate that co-facilitating such interventions with a group approach might be beneficial as patients can learn from one another’s experiences [51]. Furthermore, involving service users when planning and delivering educational group interventions provides a potential benefit to the patients and has the potential to affect service delivery and development [51–53]. However, according to recent reviews, involvement in developing interventions and research co-design is rarely evaluated empirically [43, 54, 55].

According to European mental health policy [56] and recent studies [25, 27, 28], there is an increasing need to involve service users when planning and implementing interventions in community mental health settings. In addition, a recent scoping review [43] suggested the need to use a co-facilitated approach during the delivery of the intervention, which more effectively helps to inform patients about available services outside the public healthcare system. However, to date, there are no feasibility studies evaluating psychoeducational group programmes co-facilitated and developed for adults newly diagnosed with ADHD in community mental health settings.

Feasibility studies play an important role in evaluating interventions, examining factors such as recruitment capacity, data collection procedures, the accessibility and suitability of the intervention, resource availability, and the preliminary participant response [57]. Acceptability, in turn, assesses the satisfaction and perceived appropriateness of an intervention based on expected or experienced emotional and cognitive reactions [58]. However, there is a lack of studies investigating whether patients diagnosed with ADHD in clinical settings are satisfied with psychoeducational interventions, and the acceptability of user co-facilitated interventions has yet to be investigated. To fill this gap in knowledge, we conducted a feasibility proof-of-concept randomised controlled trial study on outpatients from a Norwegian community mental health centre. The primary aim of this feasibility proof-of-concept study is to determine the feasibility and acceptability of a user co-facilitated psychoeducational programme for adults with ADHD. The secondary aim is to investigate the preliminary effects of the programme on self-efficacy, ADHD symptoms and quality of life.

## Methods

### Study design and participants

In this feasibility proof-of-concept study, we used a parallel two-arm randomised controlled trial (RCT) design with 1:1 allocation. The intervention is described according to TIDieR guidelines [59], and the report of the RCT is done according to CONSORT guidelines [60]. The psychoeducational programme took place at two centres, but due to the limited participation of user representatives one of the centres was excluded from this study [37]. Recruitment took place between November 2017 and March 2018.

### Recruitment

Patients diagnosed with ADHD who underwent outpatient treatment at the community mental health centre were eligible for inclusion. These patients were informed about the study via flyers delivered by their therapists. Patients interested in the study were contacted via mail and invited to an in-person meeting at the outpatient clinic. The inclusion criteria were as follows: age between 18 and 67 years, fluency in Norwegian, Swedish or Danish language, a confirmed diagnosis of ADHD according to DSM-IV, and willingness to participate in the study. Patients were excluded from participation if they had a psychotic disorder, severe learning difficulties, or were unable to give informed consent (i.e. the clinician deemed the patient severely cognitively impaired and unable to understand the risks and benefits of the study participation) [61]. Patients were also excluded if they were currently taking part in any other research project or had received prior group therapy for ADHD. Patients eligible for inclusion were given written and oral information about the study. Participants were required to provide written informed consent and were informed about the possibility of withdrawing their consent at any time during the study. Recruitment ended after enrolling 30 patients.

### Sample size

We determined the sample size of this feasibility study based on recommendations for pilot studies to include a minimum of 12 patients per study arm [62]. To account for an expected 25% dropout rate, we aimed to include 15 outpatients in each study arm, equal to a total sample size of 30.

### Randomisation and masking

Patients who agreed to participate were randomly assigned to an intervention (IG) or a control group (CG). Group allocation was performed via independent computer-assisted software using a block randomisation procedure (Applied Clinical Research Centre at the Norwegian University of Science and Technology). After randomisation, outpatients were informed about their group allocation. All participants received treatment as usual (TAU). The statistician (ØS) was masked at the group level when performing analyses for the primary outcome and supervising the analysis plan with pre-defined outcomes.

### Intervention and control group

#### Intervention group

The group-based psychoeducational programme was an in-person group educational programme, consisting of 10 weekly sessions (Table 1). All the participants in the IG received the intervention. We conducted three distinct groups, each comprising 9–11 study participants. Each session included a brief lecture from a recruited expert (a medical doctor, psychiatrist, psychologist, or nurse) on the session topic (20 min).

User representatives played a key role in suggesting and selecting topics for the lectures and their feedback contributed to the development of the presentation materials. These representatives, recruited from the ADHD User-led Organisation Norway (an organisation that provides support to people with ADHD), were invited to each planning meeting for this study. The selection of topics was based on the patients’ needs, as identified with the assistance of user representatives and healthcare professionals.

Each session was structured with the lecture presented first, followed by a discussion section and session closing. There was no assigned homework. After this lecture, patients were free to discuss the topics and share their experiences (45 min). The discussion section was co-facilitated by the course leader and one user representative from the ADHD User-led Organisation (acting as peer co-facilitator). The peer co-facilitator contributed by posing relevant questions and initiating discussions between the participants. The main goal of the discussion section was for the outpatients to deepen their knowledge of the implications of ADHD diagnosis by sharing their experiences and information.

The educational programme was conducted in dedicated facilities geographically separated from the main clinic, ensuring that it was isolated from the general outpatient population. This arrangement provided a quiet, distraction-free environment, exclusively for the intervention group participants (IG). Access to this room was restricted to those who were part of the intervention group.

**Table 1:** Content of the peer co-facilitated educational programme

Topic and session focus	Lecturer
Introduction Myths and facts about ADHD	Psychiatric nurse, user representative, and psychologist
What is ADHD?	Psychiatrist or psychologist and representative from ADHD Norway
Inattention	Medical doctor or psychiatrist
Impulsivity	Medical doctor or psychiatrist
Hyperactivity	Medical doctor or psychiatrist
ADHD and comorbidity	Psychiatrist or psychologist, or experienced psychiatric nurse
Use of medications	Medical doctor or psychiatrist
Economy implications	Social worker and representative from ADHD User-led Organisation in Norway
Self-help groups and coping with daily life	Representative from ADHD User-led Organisation in Norway
Work and welfare	Representative from the Norwegian Labour and Welfare Administration (NAV) Representative from ADHD Norway
Summary and closing session	Nurse and user representative from ADHD User-led Organisation in Norway

*Note* NAV is the public Norwegian labour and welfare agency

#### Control group

Outpatients in the CG received TAU consisting of medication treatment (if deemed necessary) and individual counselling or psychotherapy. Some patients also received assistance with housing, finances, support network or other important aspects of life, provided by a representative from the state social and welfare services (Norwegian Labour and Welfare Administration).

### Data collection and outcome measures

Participants from the intervention group (IG) and the control group (CG) had data collected at three time points. For IG: baseline (T0) – an informational meeting prior to randomisation, where participants got information about the study, signed written informed consent, and filled in baseline questionnaires; pre-intervention (T1) – data collection took place immediately before the intervention; post-intervention (T2) – the time point after the last lecture of the educational programme. For CG: baseline (T0) – informational meeting prior to randomisation and collecting baseline questionnaires; intermediate points T1 and T2. The level of self-efficacy, the severity of ADHD symptoms and quality of life were measured at each time point for both groups.

### Feasibility and acceptability outcomes

Feasibility was assessed for the following areas: (1) acceptance and consent to participate were determined a priori and defined as feasible if 50% of eligible outpatients accepted participation; (2) the adherence rate was predefined as the number of outpatients that attended all in-person sessions; and (3) the dropout rate was predefined as the ratio of the number of participants who dropped out of the intervention, with 25% considered an acceptable dropout rate.

Acceptability and overall patient satisfaction were assessed with the Client Satisfaction Questionnaire 4-items (CSQ-4). The CSQ-4 [63] was collected for both groups at T2. Data on this scale was collected for both the intervention group and the control group, and the results were compared. The CSQ-4 consists of four items, scored from one to four. One item is reverse-scored, and the remaining three are direct-scored. A sum of the scores between four and 16 indicates the degree of satisfaction with different aspects of the services provided. A higher total score means higher patient satisfaction. In this study, the Cronbach’s alpha was 0.85.

Patient satisfaction with the given information was assessed via three items [29] measuring what participants think of the information they received: What do you think of the information you received (a) “about ADHD”, (b) “about the treatment options”, and (c) “about pharmacological treatment?”. Each item, assessed with the 5-point Likert-type scale, was rated from one (“not satisfied”) to five (“very satisfied”). Moreover, the “I don’t know” response option was added. The maximum total score for the survey was 15, which corresponds to the highest level of satisfaction with the information. In this study, the Cronbach’s alpha was 0.76.

## Preliminary efficacy and patient-reported outcomes

### Primary outcome

#### Self-efficacy

Self-efficacy was measured by the General Self-Efficacy Scale for patients with ADHD (GSE-6-ADHD). The GSE-6 consists of six items assessed on a 4-point Likert scale ranging from one (‘not at all true’) to four (‘exactly true’), with a maximum total score of 24, which corresponds to the highest level of self-efficacy [64]. The GSE-6 has been validated among adults with ADHD. The internal consistency in the validation study was 0.91 [64]. In this study, the Cronbach’s alpha was 0.83.

### Secondary outcomes, ADHD symptoms and quality of life

Symptoms of ADHD were measured with the Hopkin’s Symptoms Checklist 9-items (SCL-9), ADHD specific subscale and the ADHD Self-Report Scale Full Edition (ASRS).

The SCL-9 consists of nine items scored from zero (“not at all”) to four (“very much”), with a maximum total score of 36, which indicates more pronounced symptoms of the disorder. This scale has been validated and tested in outpatient conditions [65]. Internal consistency in our study was 0.74 (Cronbach’s alpha). In another study the SCL-9 showed internal consistency equal 0.87 [66].

The ASRS was designed to measure symptoms in adults with ADHD [67]. The ASRS consists of 18 items and is divided into two parts: questions 1–9 reflect inattention symptoms (part A), and questions 10–18 reflect hyperactivity and impulsivity symptoms (part B). Each item was ranged from zero (“never”) to four (“very often”), with a maximum total score of 72, which indicates more pronounced symptoms. In the present study Cronbach’s alpha was .84. The internal consistency of the ASRS in other studies varied from 0.85 [68] to 0.97 [69].

Quality of life was measured using the Adult ADHD Quality of Life Scale (AAQoL) [70]. The AAQoL consists of 29 questions and four subscales. The Life Productivity subscale contains 11 items, the Psychological Health subscale contains six items, the Life Outlook subscale contains seven items, and the Relationships subscale contains five items. Items are rated on a 5-point Likert scale. The total score is the sum of all scores reversed (except the Life Outlook subscale, which is not reversed) transformed (to a 100-point scale) and divided by the number of items. The AAQoL total score ranges from zero to 100. A higher score indicates a higher quality of life (53). In the present study, Cronbach’s alpha was 0.78. The internal consistency varied from 0.74 [71] to 0.93 [72].

### Involving users’ perspectives

User representatives were actively involved in every phase of the study. During the planning and development stages of the psychoeducational programme, they were invited to identify pertinent outcomes for the RCT and choose topics for presentation within the programme. Their participation extended to the preparation of the study protocol, which included the selection of patient-reported outcomes.

One user representative played a multifaceted role, contributing to both the delivery of the intervention and the authorship of this article. During the intervention delivery, the representative led discussions, delivered portions of the lectures, and provided information about the services offered by user organisations for individuals with ADHD. They also offered support and responded to queries from study participants, maintaining this engagement even during breaks.

### Statistical analyses

SPSS was used to conduct the statistical analyses [73]. An independent statistician performed the analyses regarding primary outcomes, following the planned analyses in the study protocol without deviations. Demographic data and data from the scales were described by mean (*M*), standard deviation (*SD*), and proportions. The differences between groups for continuous data were tested using the independent samples *t*-test or Mann-Withey U test; the Chi-squared test of associations or Fisher’s exact test were used to identify differences between groups for categorical variables.

Feasibility indicators (acceptance rate, adherence rate and dropout rate) were described with absolute numbers and proportions (percentage frequencies). Satisfaction was calculated using two predefined criteria. We first calculated the total score of satisfaction, classifying it as high satisfaction if the score reached at least 75% of the maximum score (12 to16). Secondly, we calculated high satisfaction, item per item. If a patient’s score for each single item was 3 or 4 (out of 1–4), the patient was classified as satisfied with the treatment; if a patient had a score of 1 or 2, they were considered not satisfied. Rates were compared in rates. Mean group differences for patient satisfaction were also calculated using Mann-Whitney U tests (non-normally distributed data).

For the secondary outcomes, we performed an intention-to-treat analysis, with mean differences for outcomes on different time points calculated with SPSS, using linear mixed model analysis. The combination of timepoint and treatment group (with levels “Baseline”, “Pre-intervention”, “IG” and “CG”) was taken as fixed effect, while participants’ ID was taken as random effect. Next, we adjusted the model to account for the differences in employment status between the groups. Alpha levels were set to *p* < 0.05. To assess the effect size of the differences between the groups, Cohen’s *d* was calculated.

## Results

### Recruitment and feasibility

Of a total of 32 adults invited to participate, 30 outpatients were randomised. Table 2 details the demographic characteristics of the study participants by study group at baseline.

**Table 2:** Demographic and baseline characteristics

Characteristics	Total sample, *n* = 30	IG, *n* = 15	CG, *n* = 15	*p*-value
**Age**				
*M* (*SD*)	30.20 (7.941)	31.33 (8.674)	29.07 (7.255)	0.444
Range	19–47	20–47	19–41	
Missing values	2 (6.7%)	1 (6.7%)	1 (6.7%)	
**Sex**				0.330
Male	5 (16.7%)	4 (26.7%)	1 (6.7%)	
Female	25 (83.3%)	11 (73.3%)	14 (93.3%)	
**Marital status**				0.557
Single	16 (53.3%)	8 (53.3%)	8 (53.3%)	
Married	4 (13.3%)	1 (6.7%)	3 (20%)	
Live together with someone	8 (26.7%)	4 (26.7%)	4 (26.7%)	
Divorced	2 (6.7%)	2 (13.3%)	0	
**Educational level**				0.583
Primary/secondary school	7 (23.3%)	5 (33.3%)	2 (13.3%)	
Post-secondary school	18 (60.0%)	8 (53.3%)	10 (66.7%)	
High school/ university	5 (16.7%)	2 (13.3%)	3 (20.0%)	
**Employment status**				0.030
Student	8 (26.7%)	7 (46.7%)	1 (6.7%)	
Employed	9 (30.0%)	3 (20%)	6 (40%)	
Employed partly (50%)	4 (13.3%)	1 (6.7%)	3 (20.0%)	
Disabled	2 (6.7%)	2 (13.3%)	0	
Unemployed	5 (16.7%)	1 (6.7%)	4 (26.7%)	
Other	1 (3.3%)	0	1 (6.7%)	
Missing values	1 (3.3%)	1 (6.7%)	0	
**ADHD medicine**				0.598
Yes	22 (73.3%)	12 (80%)	10 (66.7%)	
No	8 (26.7%)	3 (20%)	5 (33.3%)	
**Individual counselling/psychotherapy**				1.00
Yes	16 (53.3%)	8 (53.3%)	8 (53.3%)	
No	14 (46.7%)	7 (46.7%)	7 (46.7%)	
**Comorbid diagnoses**	13 (43.3%)	7 (46.7%)	6 (40%)	1.00

*Note* ADHD = Attention deficit/hyperactivity disorder, *M* = mean, *n* = number of participants, *SD* = Standard Deviation

There were significantly more students in the IG compared with the CG. Beside this, there were no significant differences in baseline characteristics, demographic or questionnaire data between the groups.

**Fig. 1:**
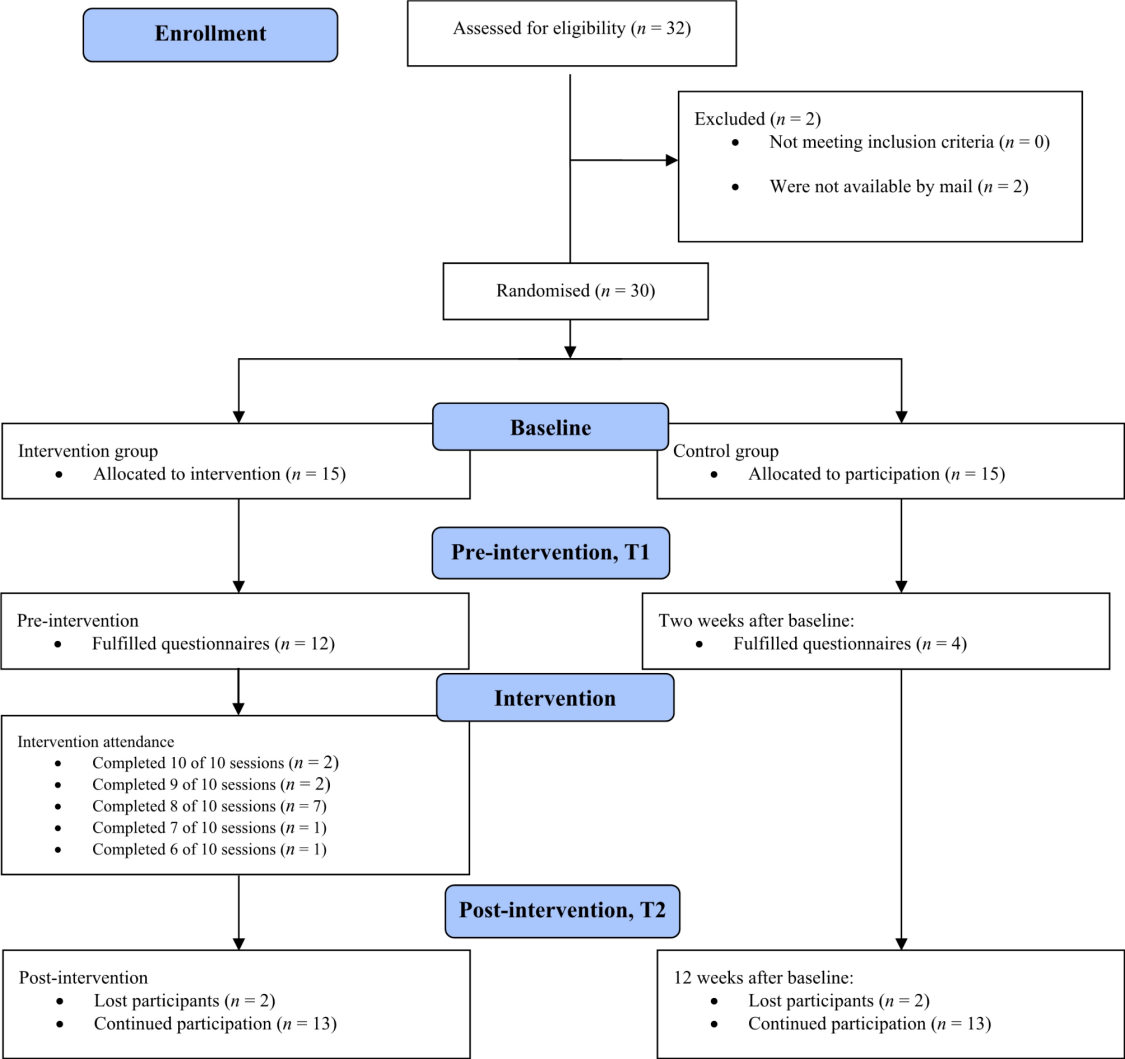
Flow of participants through the self-management group-based educational interventions study

Recruitment is presented in Fig. 1 (CONSORT flow diagram). Of the 32-invitation letters sent, two invitations were returned due to incorrect address information. This yields an overall acceptance rate of 93.8%.

### Intervention delivery

The intervention was feasible to deliver. User representatives demonstrated full participation, attending all ten sessions. They took the lead in discussions and contributed to the delivery of several lectures (refer to Table 1 for details). Two out of 15 (13.3%) participants dropped out. The overall intervention attendance rate was high, with participants attending 82.3% of the sessions. Follow-up response rates in the intervention group were high. Out of 15 participants, 12 (80%) completed questionnaires at the T1 timepoint (pre-intervention). At the T2 time point, 26 (86.6%) participants completed the questionnaires: 13 in the IG and 13 in the CG.

### Acceptability and satisfaction

Acceptability and satisfaction with the programme were examined with the CSQ-4 in T2. In total, 12 participants (80%) answered CSQ-4 in the IG and 11 (73.3%) in the CG. The between-group analyses revealed that the IG reported significantly greater satisfaction (mean CSQ-4 = 12.83, SD = 1.90) than the CG (mean CSQ-4 = 11.89, *SD* = 2.34), *p* = .027.

At T2, a significantly larger proportion of the outpatients receiving the psychoeducational intervention were classified as very satisfied: 11 out of 12 participants scored the maximum score, equal to 12 or more (91.6%), whereas 5 of 11 outpatients in the CG (45.5%), *p* = 0.027. A more detailed analysis of the item level is presented in Table 3.

**Table 3:** The proportion of participants who scored 3 or 4 on the CSQ-4 items

Items	IG (*n* = 12)	CG (*n* = 11)	*p*-value
CSQ-4 item 1 (How well has our programme met your needs)	9 (75%)	7 (63.6%)	0.667
CSQ-4 item 2 (Did the services you received help you handle problems? )	11 (91.6%)	6 (54.5%)	0.069
CSQ-4 item 3 (Would you return to our program if you needed help again)	11 (91.6%)	10 (90.9%)	1.00
CSQ-4 item 4 (Would you return to our program if you needed help again)	11 (91.6%)	7 (63.6%)	0.155

*Note* CSQ = Client Satisfaction Questionnaire

### Satisfaction with the information

As shown in Table 4, IG participants reported higher satisfaction with the information they received, as measured by the 3-item survey. Linear mixed model analyses showed statistically significant differences between groups at the T2 timepoint *p* = 0.018, 95% CI [0.472, 4.562]. Moreover, a large effect size with a Cohen’s *d* equal to 1.317 suggests a strong and significant difference between groups.

**Table 4:** Between-group comparisons of primary and secondary outcomes

Outcomes	Model-based Mean at T0	Model-based Mean at T1	Model-based Mean at T2		Model-based difference between groups at T2					
	Baseline	Pre-intervention	IG	CG	Diff	95% CI	*p*-value	*p*-value*	*p-*value for *F* test	Cohen’s *d*
GSE-6, total score	15.533	16.571	17.408	15.959	1.449	− .57, 2.954	.059	.043	.013	.461
Satisfaction with the information,3 item, total score	7.258	7.260	10.279	7.763	2.517	.472, 4.562	.018	.028	.027	1.317
SCL-9, total score	18.433	15.556	17.161	17.634	− .473	-4.496, 3.550	.814	.633	.123	.161
ASRS, total score	46.933	45.215	45.395	45.484	− .089	-4.600, 4.422	.968	.758	.442	.066
ASRS, inattention subscale	25.400	23.439	23.118	24.489	-1.37	-4.568, 1.826	.392	.330	.149	.247
ASRS, hyperactivity subscale	21.533	21.613	22.322	20.960	1.362	-1.421, 4.145	.329	.528	.926	.152
AAQoL,total score	49.51	51.884	48.933	45.699	3.235	-4.749, 11.218	.419	.270	.237	.335
AAQoL, life productivity subscale	52.417	53.139	47.804	46.106	1.698	-9.743, 13.139	.767	.488	.341	.193
AAQoL, psychological health subscale	43.611	50.677	50.038	40.611	9.427	-1.956, 20.810	.102	.114	.148	.557
AAQoL relationship subscale	51.000	59.158	59.231	46.855	12.38	2.348, 22.404	.017	.007	.012	.781
AAQoL, life outlook subscale	49.286	47.945	49.121	47.302	1.819	-4.954, 8.593	.591	.616	.847	.123

*Note:* AAQoL = Adult ADHD Quality of Life scale, ASRS = Adult ADHD Self-Reported Scale, GSE-6 = General Self-Efficacy scale, SCL-9 = Symptom-Checklist scale

* Linear mixed model, adjusted for employment status

### Preliminary effects

Preliminary results of the intervention are presented in Table 4. The initial statistical analysis did not show a statistically significant difference between groups in GSE-6 outcome (*p*-value = 0.059). However, after adjusting the linear mixed model for the employment status with the variable “employment status” as a fixed effect, the results showed statistically significant difference between the groups regarding levels of self-efficacy. The mean difference between the groups at T2 was 1.635 (*p* = .043, 95% CI [0.054, 3.216]). The linear mixed model revealed a significant improvement in the “relationship” subscale of the AAQoL for the IG versus the CG (Mean difference = 12.376, *p* = .017, 95% CI [2.348, 22.404]). There were no significant differences between groups on the other patient-reported outcomes.

## Discussion

While psychoeducational group interventions have the potential to reach many outpatients, and co-facilitating groups can have a positive impact for adults newly diagnosed with ADHD, there is a need for studies on the feasibility and potential benefits of such interventions. Based on our preliminary results, psychoeducational group programmes co-facilitated with user representatives could be both feasible and acceptable to patients and may improve patient satisfaction.

The feasibility of the programme was demonstrated in our study. The majority of the outpatients were willing to participate, with the acceptance rate in our study higher than in other peer co-facilitated studies [31, 32, 39, 74, 75]. No patients withdrew informed consent during the intervention. Moreover, 86,6% of the participants completed the intervention, comparable to previous studies [45, 46, 74, 76]. In the Hirvikoski et al., 2015 [45] study, which evaluated the “PEGASUS” psychoeducational programme, 84.3% of participants with ADHD completed the intervention. In a later study by Hirvikoski et al. (2017), 95.8% (46 out of 48) participants completed the same programme [46].

A relatively low percentage of participants dropped out in our study compared to other peer co-facilitated interventions [32, 39, 42, 74], where dropout rates were between 22% and 12.6%. Another psychoeducational programme for adults with ADHD showed comparable dropout rates [47, 76]. For instance, Hoxhaj et al. (2018) [76] compared a psychoeducational programme with a mindfulness training programme, and the dropout rate in the total sample was 7%, while in the psychoeducation group the dropout rate was 10%. Vidal et al. (2013) [47] also reported 93.75% intervention completion. The minimal dropout rate in our study may be attributed to the motivation of the patients and the active involvement of user representatives in the intervention’s development and delivery. This finding is consistent with prior research [77], which suggests that user involvement not only leads to greater study adherence, but also results in greater satisfaction of patients’ needs [78], better outcomes [55], and increased patient activation [79].

The attendance rate in our study was high, supporting the feasibility and acceptability of the intervention. Notably, only two out of 15 patients attended fewer than eight out of 10 sessions. Moreover, all the participants who completed the intervention attended more than half of the sessions. These findings are comparable to previous studies on psychoeducational programmes for adults with ADHD, where attendance rates were 84.3% [80] and 87% [81]. However, the attendance rate in our study was higher than in previous peer co-facilitated interventions. For instance, Druss et al. (2018) reported that 70% of participants attended at least four of six sessions [74], Goldberg et al. (2013) reported that 59% of participants attended five of 15 sessions [42] and Bartels et al., reported that all of the participants attended five or more out of nine sessions [82]. The high acceptance rate among newly diagnosed patients may indicate that initiating psychoeducational interventions early, when interest in understanding the disorder is at its peak, can foster greater engagement. This preliminary result could be a crucial factor to consider when designing future interventions for similar populations. Moreover, the exclusion of individuals who had previously participated in group therapy may have resulted in a selection of more engaged study participants. This suggests that interventions of this nature could benefit from targeting individuals who are newcomers to such therapeutic settings, potentially leading to increased participation. Taken together, these findings suggest that peer co-facilitated interventions in community settings could serve as a model for future interventions, emphasising the importance of collaboration with user representatives in the development of effective programmes.

Overall, the outpatients in the intervention group were highly satisfied with the programme. Compared to the CG, the IG also showed significantly higher satisfaction with the information, indicating that the patients perceived the programme in a positive way. In previous studies related to psychoeducation for ADHD, only one study [80] reported data on patient satisfaction, and participants (*n* = 41) were “willing to participate in a similar programme in the future”. In addition, a study based on peer co-facilitated intervention also reported high satisfaction with the intervention [83]. These preliminary findings suggest that outpatients, regardless of whether they were in a psychoeducational group co-facilitated by users or by healthcare professionals, reported a positive experience with psychoeducation. However, our findings should be considered preliminary as few studies have investigated patients’ satisfaction with psychoeducational programmes co-facilitated in collaboration with user representatives. Furthermore, while we used a validated scale to measure general satisfaction, the evaluation of satisfaction with the information was performed with a 3-item scale that has not been psychometrically validated in this population.

Regarding self-efficacy, the linear mixed model analysis, without adjusting for employment status, did not reveal a statistically significant difference in GSE-6 total scores between groups. There may be several reasons explaining why significant differences were not observed in this case. Firstly, the limited sample size of 13 participants who completed the GSE-6 at T2 may have left the study underpowered to detect significant differences. Secondly, our study was not specifically designed to detect effects on patient-reported outcomes, including self-efficacy. However, when adjusting the model for employment status, the results indicated a significant improvement in self-efficacy. This finding contrasts with evidence from a study on individuals with different mental disorders that found no connection between high levels of self-efficacy and maintaining employment [84]. However, given the significant role of employment status in influencing self-efficacy, future research is needed in the ADHD population. Studies could investigate how different aspects of employment (e.g., job security, job satisfaction) impact self-efficacy and related outcomes.

Regarding other patient-reported outcomes, the results of our study indicated a significant improvement in quality of life, particularly in the context of relationships. This improvement could be linked to the positive influence experienced by individuals diagnosed with ADHD when they interact with other participants and access necessary information through these educational programs. Such interactions and information can positively impact their relationships with immediate family members. However, these findings should be interpreted with caution, as our study did not indicate improvements in other aspects of quality of life. Our findings related to quality of life contrast with previous studies on group programmes for ADHD patients [85] or internet-delivered programmes [86] which reported an overall improvement in quality of life scores post-intervention. It is, however, important to note that the mean total scores at baseline in these studies were lower than ours (43.4 and 47.8 vs. 49.5, as measured using AAQoL). This lower baseline quality of life score provides a larger potential for improvement during the intervention. Additionally, our limited sample size may have restricted our ability to detect significant differences. However, regarding psychoeducational group interventions for adults diagnosed with ADHD, the current body of evidence from clinical and feasibility studies remains inconclusive concerning quality of life. For example, a feasibility study conducted in 2015 [45] demonstrated improvement in relationship quality over time. In contrast, RCTs carried out by Vidal et al. (2013) [47] and Hirvikoski et al. (2017) [46] failed to identify significant improvement. Given the relative infancy of evidence regarding psychoeducational group programmes for adults with ADHD, it becomes evident that further research, with a greater number of outpatients, is required to further investigate the impact of psychoeducational programmes on participants’ quality of life.

## Strengths and limitations

The present study has several strengths. First, this is the first intervention for adults with ADHD that has been planned and conducted in close collaboration with user representatives. In addition to a proof-of-concept RCT-design, the study was conducted in a clinical setting with high recruitment rates. The study included adults newly diagnosed with ADHD and developed a psychoeducational intervention in a context where evidence for psychoeducation group programmes remains limited.

With these strengths in mind, and based on the results of the intervention’s feasibility, future work might further explore the potential of co-facilitating psychoeducational group interventions developed in collaboration with patient organisations. This feasibility study also documented user involvement in research. However, the scarce evidence in this field regarding psychoeducational group interventions for newly diagnosed ADHD adults underscores the need for further research.

Despite these strengths, the study also has some limitations to note. As discussed, the small sample size limited our findings, and generalisability is limited as the study was conducted in one clinic only. Moreover, despite the study being an RCT, the randomisation led to an unexpected imbalance, resulting in a larger student population in the intervention group. This discrepancy was an unintended consequence of the randomisation process. It is, however, important to note that the mean baseline scores between the groups showed no significant differences. Additionally, our sample was predominantly female, comprising 83.3% of the total study sample. In comparison, other RCTs focusing on psychoeducation for adults with ADHD reported female representation ranging from 33.3 to 79.9% [43]. This gender disparity, however, mirrors the typical demographic trends observed within the Norwegian population [87]. Furthermore, although the statistical analyses were conducted by a masked independent statistician to ensure objectivity, it was impossible to mask participants due to intervention’s design. This lack of blinding may introduce bias, as participants were aware of the intervention they were receiving.

We did not collect baseline data on patient satisfaction with the treatment, which limits our ability to measure changes in satisfaction levels over time. Establishing a baseline is crucial for understanding the true impact of the intervention on patient satisfaction. Future research should incorporate designs that include multiple measurement timepoints to better capture the dynamic nature of patient satisfaction and the long-term impact of educational interventions. This approach would allow for a more comprehensive understanding of how these interventions influence patient satisfaction over time and their potential contribution to service improvements in mental health settings. To address these limitations, we recommend that further studies be conducted with baseline assessments of follow-up periods and the inclusion of baseline satisfaction data. Such studies would provide more robust evidence on the long-term benefits of educational interventions and offer valuable insights into how these interventions can enhance patient satisfaction and overall service quality in mental health care.

## Conclusions

Our proof-of-concept randomised controlled trial provides preliminary evidence supporting the feasibility and acceptability of the user co-facilitated psychoeducational programme for patients newly diagnosed with ADHD in an outpatient setting. While preliminary findings demonstrate promise in enhancing patient-reported outcomes, a more robust study with a larger sample size is essential to rigorously evaluate the intervention’s effectiveness.

## Data Availability

The datasets used during the current study can, on reasonable request, be available from the corresponding author.

## Abbreviations

AAQoL: Adult ADHD Quality of Life scale
ADHD: Attention-deficit/hyperactivity disorder
ASRS: Adult ADHD Self-Reported Scale
CG: Control group
CI: Confidence interval
CONSORT: Consolidated Standards of Reporting Trials
CSQ-4: Client Satisfaction Questionnaire 4 items
CSQ-8: Client Satisfaction Questionnaire 8 items
GSE-6: General Self Efficacy 6 abridged version for ADHD
IG: Intervention group
M: Mean
M diff: Mean difference
RCT: Randomised control trial
SCL-9: Hopkin’s Symptom Checklist 9-item scale
SD: Standard deviation
TAU: Treatment as usual

## References

[1] Kessler RC, Adler L, Barkley R, Biederman J, Conners CK, Demler O, Faraone SV, Greenhill LL, Howes MJ, Secnik K, et al. The prevalence and correlates of adult ADHD in the United States: results from the National Comorbidity Survey Replication. Am J Psychiatry. 2006;163(4):716–23. 16585449 10.1176/ajp.2006.163.4.716 PMC2859678

[2] Mannuzza S, Klein RG, Bonagura N, Malloy P, Giampino TL, Addalli KA. Hyperactive boys almost grown up. V. Replication of psychiatric status. Arch Gen Psychiatry. 1991;48(1):77–83. 1984764 10.1001/archpsyc.1991.01810250079012

[3] Weiss M, Murray C. Assessment and management of attention-deficit hyperactivity disorder in adults. CMAJ. 2003;168(6):715–22. 12642429 PMC154919

[4] Fayyad J, Sampson NA, Hwang I, Adamowski T, Aguilar-Gaxiola S, Al-Hamzawi A, Andrade LH, Borges G, de Girolamo G, Florescu S, et al. The descriptive epidemiology of DSM-IV adult ADHD in the World Health Organization World Mental Health Surveys. Atten Defic Hyperact Disord. 2017;9(1):47–65. 27866355 10.1007/s12402-016-0208-3 PMC5325787

[5] Gjervan B, Torgersen T, Nordahl HM, Rasmussen K. Functional impairment and occupational outcome in adults with ADHD. J Atten Disord. 2012;16(7):544–52. 21725028 10.1177/1087054711413074

[6] Halmoy A, Fasmer OB, Gillberg C, Haavik J. Occupational outcome in adult ADHD: impact of symptom profile, comorbid psychiatric problems, and treatment: a cross-sectional study of 414 clinically diagnosed adult ADHD patients. J Atten Disord. 2009;13(2):175–87. 19372500 10.1177/1087054708329777

[7] Kessler RC, Chiu WT, Demler O, Merikangas KR, Walters EE. Prevalence, severity, and comorbidity of 12-month DSM-IV disorders in the National Comorbidity Survey Replication. Arch Gen Psychiatry. 2005;62(6):617–27. 15939839 10.1001/archpsyc.62.6.617 PMC2847357

[8] Rasmussen K, Levander S. Untreated ADHD in adults: are there sex differences in symptoms, comorbidity, and impairment? J Atten Disord. 2009;12(4):353–60. 18367759 10.1177/1087054708314621

[9] Torgersen T, Gjervan B, Rasmussen K. ADHD in adults: a study of clinical characteristics, impairment and comorbidity. Nord J Psychiatry. 2006;60(1):38–43. 16500798 10.1080/08039480500520665

[10] Katzman MA, Bilkey TS, Chokka PR, Fallu A, Klassen LJ. Adult ADHD and comorbid disorders: clinical implications of a dimensional approach. BMC Psychiatry. 2017;17(1):302. 28830387 10.1186/s12888-017-1463-3 PMC5567978

[11] Nutt DJ, Kessler RC, Alonso J, Benbow A, Lecrubier Y, Lepine JP, Mechanic D, Tylee A. Consensus statement on the benefit to the community of ESEMeD (European Study of the Epidemiology of Mental Disorders) survey data on depression and anxiety. J Clin Psychiatry. 2007;68(Suppl 2):42–8. 17288507

[12] Adler LA, Spencer TJ, Levine LR, Ramsey JL, Tamura R, Kelsey D, Ball SG, Allen AJ, Biederman J. Functional outcomes in the treatment of adults with ADHD. J Atten Disord. 2008;11(6):720–7. 17968028 10.1177/1087054707308490

[13] Safren SA, Sprich SE, Cooper-Vince C, Knouse LE, Lerner JA. Life impairments in adults with medication-treated ADHD. J Atten Disord. 2010;13(5):524–31. 19395647 10.1177/1087054709332460 PMC3652876

[14] Barkley RA, Murphy KR. Impairment in occupational functioning and adult ADHD: the predictive utility of executive function (EF) ratings versus EF tests. Archives Clin Neuropsychology: Official J Natl Acad Neuropsychologists. 2010;25(3):157–73. 10.1093/arclin/acq014 PMC2858600 20197297

[15] Aydin Ü, Capp SJ, Tye C, Colvert E, Lau-Zhu A, Rijsdijk F, Palmer J, McLoughlin G. Quality of life, functional impairment and continuous performance task event-related potentials (ERPs) in young adults with ADHD and autism: a twin study. JCPP Adv. 2022;2(3):e12090. 37431386 10.1002/jcv2.12090 PMC10242939

[16] Newark PE, Elsässer M, Stieglitz R-D. Self-Esteem, Self-Efficacy, and resources in adults with ADHD. J Atten Disord. 2016;20(3):279–90. 23074301 10.1177/1087054712459561

[17] Newark PE, Stieglitz R-D. Therapy-relevant factors in adult ADHD from a cognitive behavioural perspective. ADHD Atten Deficit Hyperactivity Disorders. 2010;2(2):59–72. 10.1007/s12402-010-0023-1 21432591

[18] Bandura A. Self-efficacy: the exercise of control. Macmillan; 1997.

[19] Schönfeld P, Brailovskaia J, Zhang XC, Margraf J. Self-efficacy as a Mechanism Linking Daily Stress to Mental Health in students: A Three-Wave Cross-lagged Study. Psychol Rep. 2019;122(6):2074–95. 30235979 10.1177/0033294118787496

[20] Romppel M, Herrmann-Lingen C, Wachter R, Edelmann F, Düngen HD, Pieske B, Grande G. A short form of the General Self-Efficacy Scale (GSE-6): development, psychometric properties and validity in an intercultural non-clinical sample and a sample of patients at risk for heart failure. Psychosoc Med. 2013;10:Doc01. 23429426 10.3205/psm000091 PMC3578200

[21] Edel MA, Hölter T, Wassink K, Juckel G. A comparison of Mindfulness-Based Group Training and Skills Group Training in adults with ADHD. J Atten Disord. 2017;21(6):533–9. 25300813 10.1177/1087054714551635

[22] Fortuna KL, DiMilia PR, Lohman MC, Bruce ML, Zubritsky CD, Halaby MR, Walker RM, Brooks JM, Bartels SJ. Feasibility, acceptability, and preliminary effectiveness of a peer-delivered and technology supported self-management intervention for older adults with Serious Mental illness. Psychiatr Q. 2018;89(2):293–305. 28948424 10.1007/s11126-017-9534-7 PMC5874159

[23] Van Dijk S, Jeffrey J, Katz MR. A randomized, controlled, pilot study of dialectical behavior therapy skills in a psychoeducational group for individuals with bipolar disorder. J Affect Disord. 2013;145(3):386–93. 22858264 10.1016/j.jad.2012.05.054

[24] Kenter RMF, Lundervold AJ, Nordgreen T. A self-guided internet-delivered intervention for adults with ADHD: a protocol for a randomized controlled trial. Internet Interventions. 2021;26:100485. 34877262 10.1016/j.invent.2021.100485 PMC8632851

[25] Webel AR, Okonsky J, Trompeta J, Holzemer WL. A systematic review of the effectiveness of peer-based interventions on health-related behaviors in adults. Am J Public Health. 2010;100(2):247–53. 20019321 10.2105/AJPH.2008.149419 PMC2804647

[26] Kooij SJ, Bejerot S, Blackwell A, Caci H, Casas-Brugué M, Carpentier PJ, Edvinsson D, Fayyad J, Foeken K, Fitzgerald M. European consensus statement on diagnosis and treatment of adult ADHD: the European Network adult ADHD. BMC Psychiatry. 2010;10(1):67. 20815868 10.1186/1471-244X-10-67 PMC2942810

[27] Bisset M, Brown LE, Bhide S, Patel P, Zendarski N, Coghill D, Payne L, Bellgrove MA, Middeldorp CM, Sciberras E. Practitioner review: it’s time to bridge the gap - understanding the unmet needs of consumers with attention-deficit/hyperactivity disorder - a systematic review and recommendations. J Child Psychol Psychiatry. 2023;64(6):848–58. 36651107 10.1111/jcpp.13752 PMC10952204

[28] Seery C, Wrigley M, O’Riordan F, Kilbride K, Bramham J. What adults with ADHD want to know: a Delphi consensus study on the psychoeducational needs of experts by experience. Health Expect. 2022;25(5):2593–602. 35999687 10.1111/hex.13592 PMC9615057

[29] Solberg BS, Haavik J, Halmoy A. Health Care services for adults with ADHD: patient satisfaction and the role of Psycho-Education. J Atten Disord. 2019;23(1):99–108. 26088028 10.1177/1087054715587941

[30] Scholz L, Werle J, Philipsen A, Schulze M, Collonges J, Gensichen J. Effects and feasibility of psychological interventions to reduce inattention symptoms in adults with ADHD: a systematic review. J Mental Health 2020:1–14. 10.1080/09638237.2020.1818189 32954909

[31] Lara-Cabrera ML, Gjerden M, Gråwe RW, Linaker OM, Steinsbekk A. Short-term effects of a peer co-led educational programme delivered before mental health treatment: a randomised controlled trial. Patient Educ Couns. 2016;99(7):1257–61. 26905956 10.1016/j.pec.2016.02.006

[32] Lara-Cabrera ML, Salvesen Ø, Nesset MB, De las Cuevas C, Iversen VC, Gråwe RW. The effect of a brief educational programme added to mental health treatment to improve patient activation: a randomized controlled trial in community mental health centres. Patient Educ Couns. 2016;99(5):760–8. 26682971 10.1016/j.pec.2015.11.028

[33] Keen A, Lu Y, Oruche UM, Mazurenko O, Draucker CB. Activation in persons with mental health disorders: an integrative review. J Psychiatr Ment Health Nurs. 2021;28(5):873–99. 34311508 10.1111/jpm.12789

[34] Lauder K, McDowall A, Tenenbaum HR. A systematic review of interventions to support adults with ADHD at work-implications from the paucity of context-specific research for theory and practice. Front Psychol. 2022;13:893469. 36072032 10.3389/fpsyg.2022.893469 PMC9443814

[35] Bramham J, Young S, Bickerdike A, Spain D, McCartan D, Xenitidis K. Evaluation of group cognitive behavioral therapy for adults with ADHD. J Atten Disord. 2009;12(5):434–41. 18310557 10.1177/1087054708314596

[36] Nagae M, Tokunaga A, Morifuji K, Matsuzaki J, Ozawa H, Motoyama K, Honda S, Hanada H, Tanaka G, Nakane H. Efficacy of a group psychoeducation program focusing on the attitudes towards medication of children and adolescents with ADHD and their parents: a pilot study. Acta Med Nagasakiensia. 2019;62(3):77–86.

[37] Vaag JR, Lara-Cabrera ML, Hjemdal O, Gjervan B, Torgersen T. Psychoeducational groups versus waitlist in treatment of attention-deficit hyperactivity/impulsivity disorder (ADHD) in adults: a protocol for a pilot randomized waitlist-controlled multicenter trial. Pilot Feasibility Stud. 2019;5:17. 30693097 10.1186/s40814-019-0401-1 PMC6343320

[38] Lara-Cabrera ML, Salvesen O, Nesset MB, De las Cuevas C, Iversen VC, Grawe RW. The effect of a brief educational programme added to mental health treatment to improve patient activation: a randomized controlled trial in community mental health centres. Patient Educ Couns. 2016;99(5):760–8. 26682971 10.1016/j.pec.2015.11.028

[39] Koksvik JM, Linaker OM, Gråwe RW, Bjørngaard JH, Lara-Cabrera ML. The effects of a pretreatment educational group programme on mental health treatment outcomes: a randomized controlled trial. BMC Health Serv Res. 2018;18(1):665. 30157839 10.1186/s12913-018-3466-2 PMC6114285

[40] Muralidharan A, Brown CH, Li JEPEAKSMH, Walsh L, Goldberg MB. Living Well: an intervention to Improve Medical Illness Self-Management among individuals with Serious Mental illness. Psychiatr Serv. 2019;70(1):19–25. 30353790 10.1176/appi.ps.201800162 PMC6494087

[41] Higgins A, Hevey D, Boyd F, Cusack N, Downes C, Monahan M, McBennett P, Gibbons P. Outcomes of a co-facilitation skills training programme for mental health service users, family members, and clinicians: the EOLAS project. Int J Ment Health Nurs. 2018;27(2):911–21. 28994234 10.1111/inm.12388

[42] Goldberg RW, Dickerson F, Lucksted A, Brown CH, Weber E, Tenhula WN, Kreyenbuhl J, Dixon LB. Living well: an intervention to improve self-management of medical illness for individuals with serious mental illness. Psychiatr Serv. 2013;64(1):51–7. 23070062 10.1176/appi.ps.201200034 PMC8666111

[43] Pedersen H, Skliarova T, Pedersen SA, Gråwe RW, Havnen A, Lara-Cabrera ML. Psychoeducation for adult ADHD: a scoping review about characteristics, patient involvement, and content. BMC Psychiatry. 2024;24(1):73. 38273266 10.1186/s12888-024-05530-8 PMC10811906

[44] Lara-Cabrera ML, Mundal I, Schroder C, Kolltveit I, Mandahl A, Hassel AM, Torgersen T, Vaag J. The effects of peer co-led educational group intervention for adults with ADHD: preliminary results of a randomized controlled pilot study. ADHD Atten Deficit Hyperactivity Disorders. 2019;11(1):S48.

[45] Hirvikoski T, Waaler E, Lindstrom T, Bolte S, Jokinen J. Cognitive behavior therapy-based psychoeducational groups for adults with ADHD and their significant others (PEGASUS): an open clinical feasibility trial. Atten Deficit Hyperactivity Disorders. 2015;7(1):89–99. 10.1007/s12402-014-0141-2 PMC4340972 24863143

[46] Hirvikoski T, Lindstrom T, Carlsson J, Waaler E, Jokinen J, Bolte S. Psychoeducational groups for adults with ADHD and their significant others (PEGASUS): a pragmatic multicenter and randomized controlled trial. Eur Psychiatry: J Association Eur Psychiatrists. 2017;44:141–52. 10.1016/j.eurpsy.2017.04.005 28641216

[47] Vidal R, Bosch R, Nogueira M, Gomez-Barros N, Valero S, Palomar G, Corrales M, Richarte V, Mena B, Casas M, et al. Psychoeducation for adults with attention deficit hyperactivity disorder vs. cognitive behavioral group therapy: a randomized controlled pilot study. J Nerv Mental Disease. 2013;201(10):894–900. 10.1097/NMD.0b013e3182a5c2c5 24080677

[48] Haugan AJ, Sund AM, Young S, Thomsen PH, Lydersen S, Novik TS. Cognitive behavioural group therapy as addition to psychoeducation and pharmacological treatment for adolescents with ADHD symptoms and related impairments: a randomised controlled trial. BMC Psychiatry. 2022;22(1):375. 35655149 10.1186/s12888-022-04019-6 PMC9164353

[49] Skliarova T, Pedersen H, Holsbrekken Å, Pedersen SA, Mandal A, De Las Cuevas C, Havnen A, Gråwe R, Lara-Cabrera ML. Psychoeducational group interventions for adults diagnosed with attention-deficit/ hyperactivity disorder: a scoping review of feasibility, acceptability, and outcome measures. BMC Psychiatry. 2024;24(1):463. 38902683 10.1186/s12888-024-05908-8 PMC11191191

[50] Abadi MH, Barker AM, Rao SR, Orner M, Rychener D, Bokhour BG. Examining the impact of a Peer-Led Group Program for veteran Engagement and Well-Being. J Altern Complement Med. 2021;27(S1):S37–44. 33788603 10.1089/acm.2020.0124

[51] Cooper RE, Saunders KRK, Greenburgh A, Shah P, Appleton R, Machin K, Jeynes T, Barnett P, Allan SM, Griffiths J, et al. The effectiveness, implementation, and experiences of peer support approaches for mental health: a systematic umbrella review. BMC Med. 2024;22(1):72. 38418998 10.1186/s12916-024-03260-y PMC10902990

[52] Matthews EB, Rahman R, Schiefelbein F, Galis D, Clark C, Patel R. Identifying key roles and responsibilities of peer workers in behavioral health services: a scoping review. Patient Educ Couns. 2023;114:107858. 37348313 10.1016/j.pec.2023.107858

[53] Åkerblom KB, Ness O. Peer workers in co-production and co-creation in Mental Health and Substance Use Services: a scoping review. Adm Policy Ment Health. 2023;50(2):296–316. 36396756 10.1007/s10488-022-01242-x PMC9931804

[54] Slattery P, Saeri AK, Bragge P. Research co-design in health: a rapid overview of reviews. Health Res Policy Syst. 2020;18(1):17. 32046728 10.1186/s12961-020-0528-9 PMC7014755

[55] Ezaydi N, Sheldon E, Kenny A, Buck ET, Weich S. Service user involvement in mental health service commissioning, development and delivery: a systematic review of service level outcomes. Health Expect. 2023;26(4):1453–66. 37292036 10.1111/hex.13788 PMC10349231

[56] Kooij JJS, Bijlenga D, Salerno L, Jaeschke R, Bitter I, Balazs J, Thome J, Dom G, Kasper S, Nunes Filipe C, et al. Updated European Consensus Statement on diagnosis and treatment of adult ADHD. Eur Psychiatry: J Association Eur Psychiatrists. 2019;56:14–34. 10.1016/j.eurpsy.2018.11.001 30453134

[57] Orsmond GI, Cohn ES. The distinctive features of a feasibility study:objectives and guiding questions. OTJR: Occup Therapy J Res. 2015;35(3):169–77. 10.1177/1539449215578649 26594739

[58] Sekhon M, Cartwright M, Francis JJ. Acceptability of healthcare interventions: an overview of reviews and development of a theoretical framework. BMC Health Serv Res. 2017;17(1):88. 28126032 10.1186/s12913-017-2031-8 PMC5267473

[59] Gupta D, Rao A, Shenoy R, BS S. TIDieR-Checklist. In.: figshare; 2022.

[60] Schulz KF, Altman DG, Moher D, the CG. CONSORT 2010 Statement: updated guidelines for reporting parallel group randomised trials. BMC Med. 2010;8(1):18. 20334633 10.1186/1741-7015-8-18 PMC2860339

[61] Peter Bates CW. How to gain informed consent. In; 2020: 10.

[62] Julious SA. Sample size of 12 per group rule of thumb for a pilot study. Pharm Stat. 2005;4(4):287–91. 10.1002/pst.185

[63] Pedersen H, Skliarova T, Attkisson CC, Lara-Cabrera ML, Havnen A. Measuring patient satisfaction with four items: validity of the client satisfaction questionnaire 4 in an outpatient population. BMC Psychiatry. 2023;23(1):808. 37936112 10.1186/s12888-023-05310-w PMC10630992

[64] Skliarova T, Pedersen H, Hafstad H, Vaag JR, Lara-Cabrera ML, Havnen A. The construct validity of an abridged version of the general self-efficacy scale for adults with attention-deficit/hyperactivity disorder. Front Psychiatry 2023, 14. 10.3389/fpsyt.2023.1212961 PMC10657811 38025439

[65] Eich D, Angst J, Frei A, Ajdacic-Gross V, Rössler W, Gamma A. A new rating scale for adult ADHD based on the Symptom Checklist 90 (SCL-90-R). Eur Arch Psychiatry Clin NeuroSci. 2012;262:519–28. 22212725 10.1007/s00406-011-0288-1

[66] Prinz U, Nutzinger DO, Schulz H, Petermann F, Braukhaus C, Andreas S. Comparative psychometric analyses of the SCL-90-R and its short versions in patients with affective disorders. BMC Psychiatry. 2013;13(1):104. 23537095 10.1186/1471-244X-13-104 PMC3626675

[67] Kessler RC, Adler L, Ames M, Demler O, Faraone S, Hiripi E, Howes MJ, Jin R, Secnik K, Spencer T, et al. Scale (ASRS): a short screening scale for use in the general population. Psychol Med. 2005;35(2):245–56. The World Health Organization Adult ADHD Self-Report. 15841682 10.1017/S0033291704002892

[68] Zohar AH, Konfortes H. Diagnosing ADHD in Israeli adults: the psychometric properties of the adult ADHD Self Report Scale (ASRS) in Hebrew. Isr J Psychiatry Relat Sci. 2010;47(4):308–15. 21270505

[69] Brevik EJ, Lundervold AJ, Haavik J, Posserud MB. Validity and accuracy of the adult Attention-Deficit/Hyperactivity disorder (ADHD) self-report scale (ASRS) and the Wender Utah rating scale (WURS) symptom checklists in discriminating between adults with and without ADHD. Brain Behav. 2020;10(6):e01605. 32285644 10.1002/brb3.1605 PMC7303368

[70] Gjervan B, Torgersen T, Hjemdal O. The Norwegian translation of the adult Attention-Deficit/Hyperactivity disorder quality of Life Scale: Validation and Assessment of QoL in 313 adults with ADHD. J Atten Disord. 2019;23(9):931–9. 27033881 10.1177/1087054716640087

[71] Brod M, Adler LA, Lipsius S, Tanaka Y, Heinloth AN, Upadhyaya H. Validation of the adult attention-deficit/hyperactivity disorder quality-of-life scale in European patients: comparison with patients from the USA. Atten Defic Hyperact Disord. 2015;7(2):141–50. 25563210 10.1007/s12402-014-0160-z PMC4449381

[72] Brod M, Johnston J, Able S, Swindle R. Validation of the adult attention-deficit/hyperactivity disorder quality-of-life scale (AAQoL): a disease-specific quality-of-life measure. Qual Life Res. 2006;15(1):117–29. 16411036 10.1007/s11136-005-8325-z

[73] SPSS I. IBM SPSS statistics for Windows, version 20.0. New York: IBM Corp; 2011.

[74] Druss BG, Singh M, von Esenwein SA, Glick GE, Tapscott S, Tucker SJ, Lally CA, Sterling EW. Peer-led self-management of General Medical conditions for patients with Serious Mental illnesses: a Randomized Trial. Psychiatric Serv. 2018;69(5):529–35. 10.1176/appi.ps.201700352 PMC5930018 29385952

[75] Druss BG, Zhao L, von Esenwein SA, Bona JR, Fricks L, Jenkins-Tucker S, Sterling E, DiClemente R, Lorig K. The health and recovery peer (HARP) program: a peer-led intervention to improve medical self-management for persons with serious mental illness. Schizophr Res. 2010;118(1):264–70. 20185272 10.1016/j.schres.2010.01.026 PMC2856811

[76] Hoxhaj E, Sadohara C, Borel P, D’Amelio R, Sobanski E, Muller H, Feige B, Matthies S, Philipsen A. Mindfulness vs psychoeducation in adult ADHD: a randomized controlled trial. Eur Archives Psychiatry Clin Neurosci. 2018;268(4):321–35. 10.1007/s00406-018-0868-4 29356899

[77] Killikelly C, He Z, Reeder C, Wykes T. Improving adherence to web-based and Mobile technologies for people with psychosis: systematic review of new potential predictors of adherence. JMIR Mhealth Uhealth. 2017;5(7):e94. 28729235 10.2196/mhealth.7088 PMC5544896

[78] Dengsø KE, Lindholm ST, Herling SF, Pedersen M, Nørskov KH, Collet MO, Nielsen IH, Christiansen MG, Engedal MS, Moen HW, et al. Patient and public involvement in nordic healthcare research: a scoping review of contemporary practice. Res Involv Engagem. 2023;9(1):72. 37649111 10.1186/s40900-023-00490-x PMC10466765

[79] Moljord IE, Helland-Hansen KA, Salvesen Ø, Olsø TM, Gudde CB, Rise MB, Steinsbekk A, Eriksen L. Short time effect of a self-referral to inpatient treatment for patients with severe mental disorders: a randomized controlled trial. BMC Health Serv Res. 2016;16(1):513. 27659102 10.1186/s12913-016-1712-z PMC5034559

[80] Hirvikoski T, Waaler E, Lindström T, Bölte S, Jokinen J. Psychoeducational groups for adults with ADHD and their significant others (PEGASUS): an open clinical feasibility trial. ADHD Atten Deficit Hyperactivity Disorders. 2015;7:S94. 10.1007/s12402-014-0141-2 PMC4340972 24863143

[81] Hartung CM, Canu WH, Serrano JW, Vasko JM, Stevens AE, Abu-Ramadan TM, Bodalski EA, Neger EN, Bridges RM, Gleason LL et al. A New Organizational and Study Skills Intervention for College Students with ADHD. *Cognitive and behavioral practice* 2020.

[82] Bartels SJ, Aschbrenner KA, Rolin SA, Hendrick DC, Naslund JA, Faber MJ. Activating older adults with serious mental illness for collaborative primary care visits. Psychiatr Rehabil J. 2013;36(4):278–88. 24219769 10.1037/prj0000024 PMC4994809

[83] Green CA, Janoff SL, Yarborough BJH, Paulson RI. The Recovery Group Project: development of an intervention led jointly by peer and professional counselors. Psychiatric Serv. 2013;64(12):1211–7. 10.1176/appi.ps.201200546 23999845

[84] Waynor WR, Gill KJ, Gao N. The role of work related self-efficacy in supported employment for people living with serious mental illnesses. Psychiatr Rehabil J. 2016;39(1):62–7. 26461435 10.1037/prj0000156

[85] Carroll P, Hirvikoski T, Lindholm C, Thorell LB. Group-based emotion regulation skills training for adults with ADHD: a feasibility study in an outpatient psychiatric setting. Appl Neuropsychology: Adult. 2023;30(1):71–82. 10.1080/23279095.2021.1910512 33905287

[86] Kenter R, Gjestad R, Lundervold A, Nordgreen T. A self-guided internet-delivered intervention for adults with ADHD: results from a randomized controlled trial. Internet Interventions. 2023;32:100614. 36969389 10.1016/j.invent.2023.100614 PMC10033990

[87] Pedersen PB, Lilleeng S. E.: Distriktspsykiatriske tjenester – Driftsindikatorer for distriktspsykiatriske sentre 2017. In. Edited by Helsedirektoratet; 2019.

